# Controlled release microspheres loaded with BMP7 suppress primary tumors from human glioblastoma

**DOI:** 10.18632/oncotarget.3459

**Published:** 2015-03-26

**Authors:** P. González-Gómez, J. Crecente-Campo, C. Zahonero, M. de la Fuente, Aurelio Hernández-Laín, H. Mira, P. Sánchez-Gómez, M. Garcia-Fuentes

**Affiliations:** ^1^ UFIEC, Instituto de Salud Carlos III, Majadahonda, Madrid, Spain; ^2^ Center for Research in Molecular Medicine and Chronic Diseases (CIMUS), Dep. Pharmacy and Pharmaceutical Technology, and Health Research Institute (IDIS), Universidad de Santiago de Compostela, Santiago de Compostela, A Coruña, Spain; ^3^ Nano-oncologicals Laboratory, Translational Medical Oncology, Clinical University Hospital and Health Research Institute of Santiago de Compostela (IDIS), Santiago de Compostela, Spain; ^4^ Unidad Multidisciplinar de Neurooncología, Hospital Universitario 12 de Octubre, Madrid, Spain

**Keywords:** bone morphogenetic protein, glioblastoma, tumor initiating cells, microspheres, controlled release

## Abstract

Glioblastoma tumor initiating cells are believed to be the main drivers behind tumor recurrence, and therefore therapies that specifically manage this population are of great medical interest. In a previous work, we synthesized controlled release microspheres optimized for intracranial delivery of BMP7, and showed that these devices are able to stop the *in vitro* growth of a glioma cell line. Towards the translational development of this technology, we now explore these microspheres in further detail and characterize the mechanism of action and the *in vivo* therapeutic potential using tumor models relevant for the clinical setting: human primary glioblastoma cell lines. Our results show that BMP7 can stop the proliferation and block the self-renewal capacity of those primary cell lines that express the receptor *BMPR1B*. BMP7 was encapsulated in poly (lactic-co-glycolic acid) microspheres in the form of a complex with heparin and Tetronic, and the formulation provided effective release for several weeks, a process controlled by carrier degradation. Data from xenografts confirmed reduced and delayed tumor formation for animals treated with BMP7-loaded microspheres. This effect was coincident with the activation of the canonical BMP signaling pathway. Importantly, tumors treated with BMP7-loaded microspheres also showed downregulation of several markers that may be related to a malignant stem cell-like phenotype: CD133^+^, Olig2, and GFAPδ. We also observed that tumors treated with BMP7-loaded microspheres showed enhanced expression of cell cycle inhibitors and reduced expression of the proliferation marker PCNA. In summary, BMP7-loaded controlled release microspheres are able to inhibit GBM growth and reduce malignancy markers. We envisage that this kind of selective therapy for tumor initiating cells could have a synergistic effect in combination with conventional cytoreductive therapy (chemo-, radiotherapy) or with immunotherapy.

## INTRODUCTION

A glioblastoma (GBM) is a highly aggressive brain tumor characterized by its lack of response to conventional chemo-, radio-, and immunotherapies. Recent studies suggest that recurrence of GBM may be due to the therapeutic resistance of a subpopulation of undifferentiated GBM tumor-initiating cells (GBM-TICs). Consequently, it has been proposed that a potential treatment for GBM is to induce differentiation of GBM-TICs to a more benign phenotype or to a cell type more amenable to standard therapies [1–3].

Bone morphogenetic proteins (BMPs) are among the most potent inducers of GBM-TIC differentiation. Indeed, BMPs have been considered non-cytotoxic therapeutic compounds that may be of use in preventing the growth and recurrence of GBM [4]. BMPs signal via specific serine/threonine kinase receptors on the cell surface known as bone morphogenetic protein receptors (BMPRs). Both type-I and -II BMPRs are required for signal transduction. The type-I receptor BMPRIA preferentially binds ligands of the Dpp class, such as BMP2 and BMP4, whereas the type-I receptor BMPRIB binds ligands of the 60A class, such as BMP7. After ligand binding, the type-I/type-II receptor complex phosphorylates Smad1, 5 and 8 proteins that translocate to the nucleus to regulate target gene expression [5]. Previous studies have shown that treatment of GBM-TICs with BMP4 or BMP7 triggers Smad-mediated signaling, leading to the inhibition of cell proliferation, the induction of differentiation and a reduction of tumor formation in immunodeficient mice [4, 6, 7]. In a minority of GBMs displaying epigenetic silencing of the type I receptor BMPRIB, however, BMPs fail to induce differentiation but instead support the proliferation of GBM-TICs, thereby promoting tumorigenesis [8].

Despite this promising pharmacological profile, any attempt to use soluble BMPs as a GBM-TICs suppressor will face important shortcomings due to its fast *in vivo* erosion [9], and its incapacity to cross the blood-brain barrier [10]. To address this problem, we have recently reported the preparation of a microsphere formulation optimized for loading BMP7, and intended as an intracranial sustained release implant for managing GBM-TICs upon primary tumor resection [11]. These microspheres were able to release BMP7 for more than two months, and this released protein was able to stop the *in vitro* growth of a glioma cell line model. Although this cell line model (U87MG spheres) was interesting for screening purposes, however, the results were admittedly far from indicative of the therapeutic potential, and inadequate for analyzing the mechanism of action of the therapeutic device.

In this work, we provide further characterization of the structure and release mechanism of the microspheres, and we test the potential spectrum of applicability of our therapeutic device, its *in vivo* therapeutic potential, and mechanism of action. These activity/mechanistic studies were performed with primary GBM-TICs cultures from human glioblastoma, a clinically applicable model for addressing such questions.

## RESULTS

### BMP7 inhibits the sphere formation capacity of GBM-TICs expressing BMPR1B

**Figure 1:**
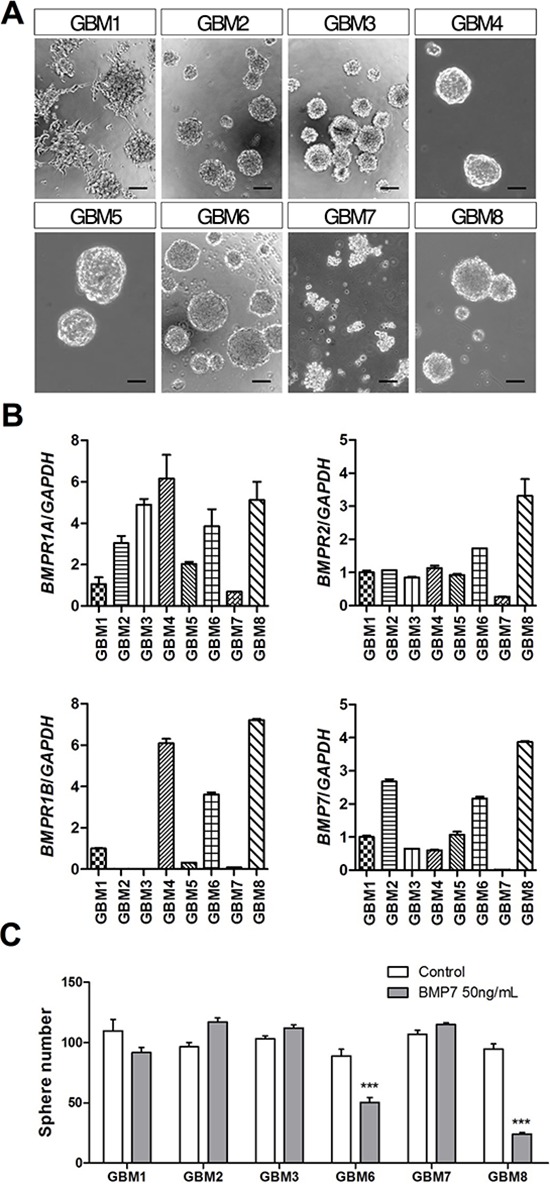
BMP7 decreases sphere formation in primary GBM-TIC cultures expressing *BMPR1B* **(A)** Representative phase-contrast images of the different GBM-TIC cultures used in the study. Scale bar: 100 μm. **(B)** *BMPR1A*, *BMPR1B*, *BMPR2* and *BMP7* relative mRNA expression levels of the cultures. GAPDH was used as the RT-qPCR housekeeping gene. **(C)** Quantification of the GBM-TIC culture capacity to form spheres in the presence/absence of 50 ng/mL of BMP7. Cells were seeded at low density in 96-well plates and spheres were counted after 7 days. ***p < 0.001.

Increasing evidence strongly supports a key role for BMP7 as a glioma growth inhibitor. In most tumors, treatment of GBM-TICs with BMP7 inhibits proliferation and promotes differentiation [4]; however, in a minority of GBMs, BMPs fail to induce differentiation of GBM-TICs due to epigenetic silencing of *BMPR1B* [8]. In order to further define the effect of BMP7 on gliomagenesis, we tested 8 primary GBM-TIC cell lines (GBM1–8) established in our laboratory from surgically resected human specimens of adult GBM patients. GBM1–8 primary cell lines grow in suspension as non-adherent spheres and proliferate in serum-free media supplemented with EGF and bFGF (Figure 1A).

Since previous studies using GBM-TICs have shown tumor-to-tumor variation in the expression of BMP receptors, we first determined the mRNA levels of *BMP7, BMPR1A, BMPR1B* and *BMPR2* genes by real-time qRT-PCR in our primary cell lines. We found that *BMPR2* and *BMPR1A* were expressed in all the GBM-TICs, albeit at different levels. *BMP7* itself was expressed in all but one sample (GBM7), whereas *BMPR1B* was only significantly expressed in three cultures: GBM4, GBM6 and GBM8 (Figure 1B). Consistently, only GBM6 and GBM8 (GBM4 could not be tested) were able to respond to BMP7 by diminishing their clonogenic capacity in a sphere formation assay (Figure 1C). The most sensitive cell line, GBM8, expressed the highest levels of the BMP receptor genes and was selected to pursue our study.

### BMP7 reduces GBM-TIC proliferation, clonogenicity and self-renewal capacity

**Figure 2:**
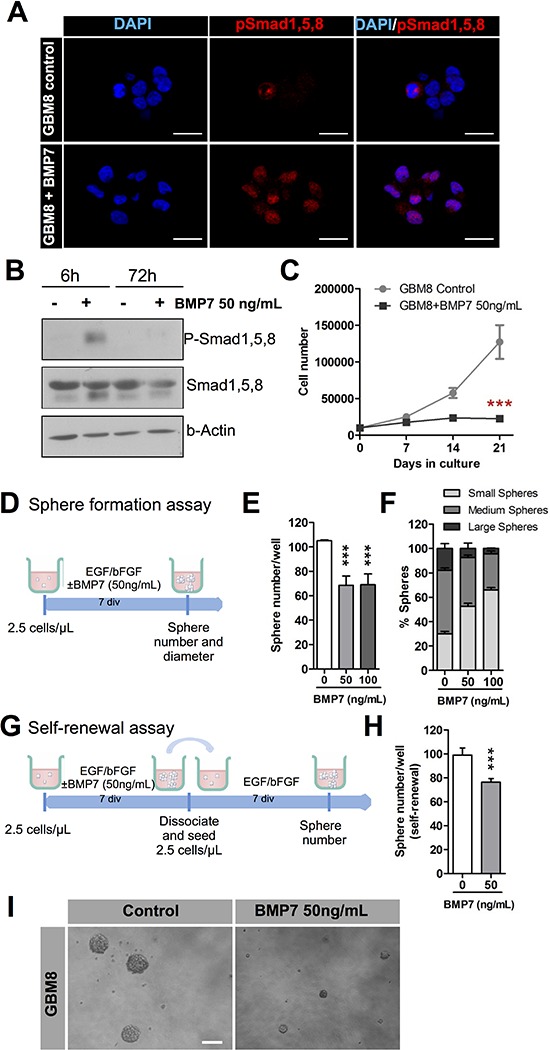
BMP7 decreases cell growth, sphere formation and self-renewal and it exerts its action via canonical signaling **(A)** Immunocytochemistry analysis of p-Smad1, 5, 8 location (red) after 6 h treatment of the culture with 50ng/mL of BMP7. DAPI stain is shown in blue. **(B)** Western blot analysis of p-Smad1, 5, 8 and total Smad1, 5, 8 after addition of 50ng/mL of BMP7 to the GMB culture. β-actin expression was used as the loading control. **(C)** Long-term growth curve of GBM8-TIC culture expanded *in vitro* over 3 weeks (*n* = 3). **(D–F)** Sphere formation assay. (D) Diagram representing the strategy used to assess the effect of BMP7 on the sphere formation capacity of the GBM culture in the absence or in the presence of two different BMP7 concentrations (*n* = 3) (E) Quantification of the number of spheres. (F) Percentage of small, medium and large spheres that appear in each condition. **(G–H)** Self-renewal assay. Quantification of the GBM-TIC capacity to form secondary spheres after BMP7 removal. (G) Schematic representation of the assay timeline. (H) Number of secondary spheres. **(I)** Representative image of a sphere formation assay. ***p < 0.001. Scale bar in A: 25 μm. Scale bar in I: 100 μm.

As a consequence of having functional receptors, BMP7 induced canonical BMP signaling in GBM8, as evidenced by both their translocation to the nucleus (Figure 2A) and their transient phosphorylation of the Smad1, 5, 8 effectors at 6 h (Figure 2B). The presence of BMP7 severely impaired long-term expansion of GBM8 sphere cultures, as we saw from the sphere cultures expanded *in vitro* for three cell passages (21 days, Figure 2C).

We then performed a sphere formation assay on GBM8 cells seeded at low density in the presence of increasing doses of BMP7 (Figure 2D). We found a decrease in the number of BMP7 treated spheres compared to the control group (Figure 2E). The size of the spheres derived was also decreased (Figure 2F). This *in vitro* phenotype reflects the inhibited proliferative activity of the GBM-TICs in the presence of 50 ng/mL and 100 ng/mL of BMP7. To determine whether BMP7 regulates GBM-TIC self-renewal, spheres that had been grown in BMP7 were dissociated and plated in the presence of mitogens but without the BMP ligand (Figure 2G). As a control, the same experiment was performed with cells not pretreated with BMP7. As shown in Figure 2H and 2I, the number of spheres that were able to grow in BMP7 pretreated cultures was significantly lower, indicating that cells had been switched to a phenotype with decreased self-renewal capacity.

### Microspheres with BMP7 prepared using a nanocomplex encapsulation method

Previous studies have shown that BMP7 can be incorporated into poly(lactic-co-glycolic) (PLGA) microspheres in the form of a heparin-Tetronic complex, a method named nanocomplex encapsulation. This method results in high protein encapsulation and controlled release properties for several weeks [11]. We selected this formulation for the *in vivo* experiments (Section 3.4), and we characterized the microsphere system in further detail.

**Figure 3:**
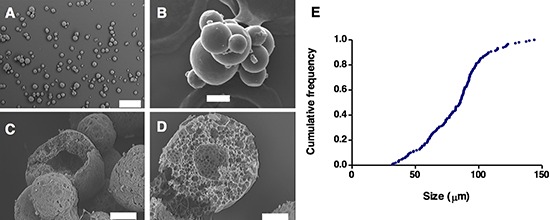
Microsphere morphology and structure. Scanning electron microscopy images of microsphere populations (A, C), and individual microspheres (B, D) Images of microspheres after preparation **(A, B)**, and after partial matrix hydrolysis in PBS at 37°C **(C, D)**. Graph **(E)** is an accumulated frequency plot of the microsphere particle size distribution.

Microspheres loaded with BMP7 presented a spherical morphology, regular surface, and absence of matrix pores (Figure 3A, 3B). Incubation of the microspheres under hydrolytic conditions resulted in particle breakup, and disclosed a frequently hollow inner structure (Figure 3C, 3D). Particle size followed a normal distribution (r^2^=0.987), with an average of 81 ± 24 μm (Figure 3E). Microsphere yield was 93.5 ± 5.1%. FTIR analysis of the microspheres showed a spectrum consistent with a mixed PLGA/Tetronic composition. A redshift of the hydroxide band, between 3455 cm^−1^ (PLGA), 3509 cm^−1^ (Tetronic) and 3397 cm^−1^ (microspheres) suggested a non-covalent interaction between these two materials (results not shown).

**Figure 4:**
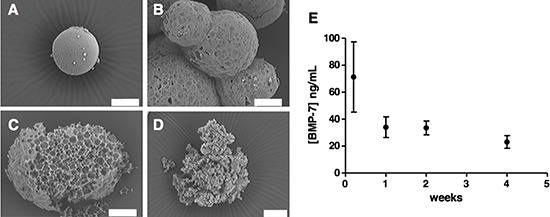
Microsphere morphology and structure during degradation in PBS (pH 7.4) BSA 1% (w/v) at 37°C Images taken at the starting point of the experiment **(A)**, after 1 week **(B)**, 2 weeks **(C)** and 4 weeks **(D)**. **(E)** BMP7 concentration released at these time points (distributive data) was measured by ELISA.

We then characterized microsphere erosion under release conditions (PBS 1% BSA, 37°C) to understand its role in BMP7 release. SEM images showed microsphere erosion over a period of 4 weeks. At the starting point the microspheres were characterized by a regular and flat surface (Figure 4A). At one week, signs of pore formation were evident on the particle surface (Figure 4B). By two weeks, particle breakup was observed, revealing a porous inner structure (Figure 4C), and frequently hollow inner structures (Figure 3C, 3D). By four weeks, particle structure had often collapsed completely (Figure 4D). The microspheres released BMP7 at all the time points studied by SEM (Figure 4E), confirming more than 4 weeks of sustained protein release.

### BMP7-loaded microspheres act as a glioma tumor suppressor *in vivo*

**Figure 5:**
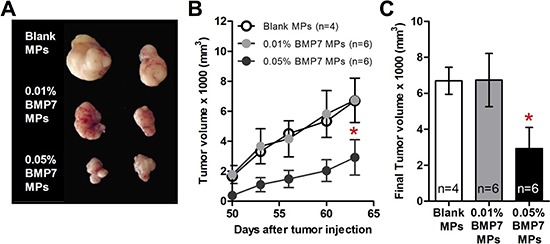
BMP7 released from the particles decreased the xenograft growth of GBM cells in the athymic nude mice Microspheres (MPs) were loaded with heparin (Blank), 0.01% (w/w) BMP7 or 0.05% (w/w) BMP7. The sample value (n) for each group was Blank *n* = 4, 0.01% BMP7 *n* = 6 and 0.05% BMP7 *n* = 6. **(A)** Representative picture of the tumors on the last day of the experiment. Not all the mice were alive at the end of the experiment for the picture. **(B)** Graph represents the tumor volume measured every 4–5 days. **(C)** Final tumor volume measurements. *p < 0.05.

In order to test the effect of BMP7 on tumor growth, 1.5 × 10^6^ cells mixed with microspheres (blank, 0.01% BMP7, and 0.05% BMP7 loading) were subcutaneously transplanted into nude mice flanks. The tumor sizes were measured from the moment they were detectable in order to calculate their growth curves. While control tumors and 0.01% BMP7 microsphere treated tumors were of similar size, the 0.05% BMP7 microsphere treated tumors presented a reduced volume upon extraction at the endpoint of the experiment (Figure 5A and 5B), and an impaired growth rate (Figure 5C).

**Figure 6:**
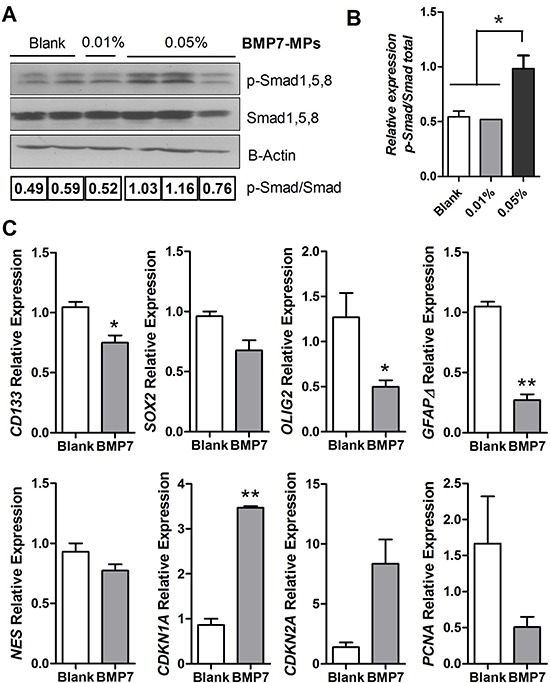
BMP signaling and gene expression analysis of the GBM xenografts **(A)** Western blot analysis of p-Smad1, 5, 8 and total Smad1, 5, 8 in the tumors at the end of the experiment. β-actin was used as the loading control. **(B)** Western blot quantification, the amount of p-Smad1, 5, 8 is shown relative to the amount of total-Smad1, 5, 8. *n* = 3 independent tumors. **(C)** *CD133*, *SOX2*, *OLIG2*, *GFAPδ*, *NESTIN*, and *CDKN1A, CDKN2A and PCNA* relative mRNA expression levels of the tumor tissue. *GAPDH* was used as the RTqPCR housekeeping gene. *n* = 3 independent tumors. *p < 0.05, **p < 0.01.

To further confirm the effect of BMP7 on tumor cells *in vivo*, the downstream signaling activation of Smad1, 5, 8 was analyzed by western blot using a specific antibody against the phosphorylated protein. Tumors implanted with 0.05% BMP7 microspheres showed higher activation of the canonical BMP route (Figure 6A and 6B). We also explored whether BMP7 signaling in tumors lead to the differentiation of tumor initiating cells, as suggested by other authors. We did not find specific differentiation genes upregulated at the mRNA level (GFAPα or Tubb3, data not shown), but we confirmed a downregulation of stem cell markers such as *SOX2*, *CD133*, *NESTIN, OLIG2* and *GFAPδ*. Consistently, we observed overexpression of the cell cycle negative regulators *CDKN1A* and *CDKN2A*, and downregulation of the proliferation cell marker *PCNA* (Figure 6C).

**Figure 7:**
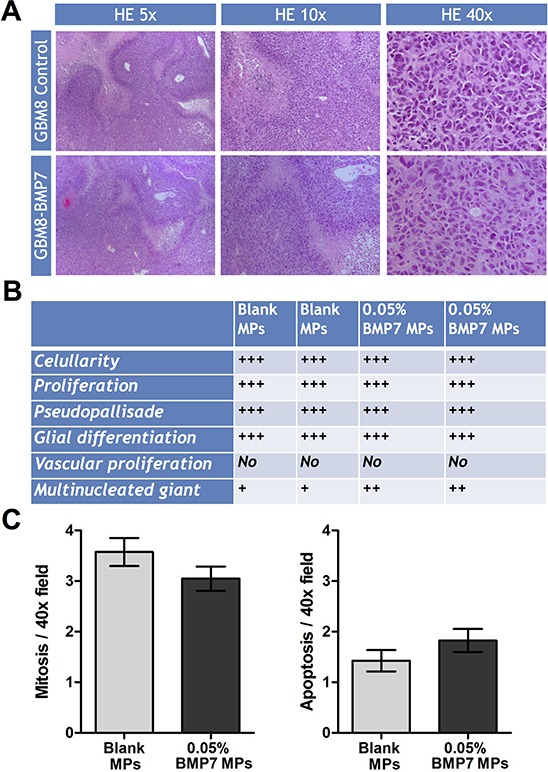
Histologic analysis of the GBM xenograft tumors treated with or without BMP7 **(A)** Representative hematoxylin and eosin (H&E) images of paraffin-embedded tumor tissue sections. 5X, 10X and 40X images are shown. **(B)** Estimation of the amount of cellularity, proliferation, pseudopallisade necrosis, glial differentiation, vascular proliferation and multinucleated giant cells. **(C)** Quantification of mitosis: number of mitosis figures per 40X field (average ±SEM of 10 fields). Quantification of apoptosis: number of apoptosis figures per 40X field (average ±SEM of 10 fields).

We then analyzed the histology of the tumors by staining tumor tissue sections with hematoxilin-eosin (Figure 7A). Although the size of the tumors was smaller in the BMP7 treated mice, we found no difference at the cellular level. All the tumors had the same rate of cellularity, glial differentiation, and vascular proliferation, and moreover, they all formed pseudopallisade necrosis (Figure 7B). When we quantified the number of mitosis and apoptosis per 40x field, the differences between control and treated tumors were not statistically significant (Figure 7C).

## DISCUSSION

Despite recent progress in diagnostic and therapeutic strategies, GBM remains one of the most lethal human tumors due to inevitable recurrence [14, 15]. One of the obstacles to developing effective therapies for GBM patients arises from the diversity of glioblastoma cell populations. Cellular heterogeneity mainly results from the stepwise accumulation of genetic alterations, and from the existence of a hierarchical tumor cell organization that includes stem-like cells (the tumor-initiating cells, TICs), precursors and differentiated cells [16]. Among the diverse GBM cell types, TICs are likely responsible for rapid tumor relapse given that they are radiation and chemotherapy resistant. Consequently, it has been widely suggested that forcing differentiation of stem-like GBM-TICs could be beneficial in stopping tumor growth or making the tumor more sensitive to standard therapies [1, 2, 8, 17].

The developmental pathways implicated in stem cell differentiation can be exploited in order to induce differentiation of GBM-TICs. Among these pathways, BMPs have a large therapeutic potential given that they have been previously linked to tumor suppression in glial tumors. It has been reported that: (1) BMP ligands are differentially expressed in tumor *versus* healthy tissue with a neat clinical relevance [18, 19]; (2) activation of the BMP pathway reduces glioma cell proliferation *in vitro* and *in vivo* and induces differentiation of the GBM-TICs [4, 7, 20–22] and (3) BMPs render GBM-TICs more susceptible to conventional therapy [23, 24] (for a review see [25]).

In the low cell density *in vitro* assays, our data clearly shows that canonical signaling downstream of BMP7 decreases GBM-TIC self-renewal and proliferation, which is in accordance with previous studies [6, 7, 21]. We also show that the expression of *BMPR1B* is essential for the responsiveness of the cells. A recent report [9] showed that 20% of GBM tumors have CpG methylation in the *BMPR1B* promoter, which results in its epigenetic silencing. In primary human GBM-TICs without *BMPR1B* expression, BMP treatment increased proliferation while forced expression of *BMPR1B* restored the normal differentiation capacity of the TICs. In our study, only 2 out of 8 primary GBM-TICs cultures (25%) responded to BMP7 treatment, and those were the ones with a high expression of *BMPR1B* receptor. Contrary to Lee et al. [9], we did not observe over-proliferation in response to BMP7 in those GBM-TIC cultures with no detectable levels of *BMPR1B* expression. This difference might be related to the different morphogen investigated in this study and our own (BMP4 vs. BMP7), and their transduced signal upon BMPR1A binding.

Despite the interesting pharmacological action of BMP7, its medical use in GBM is practically limited by an unfavorable biopharmaceutical profile. We have recently reported the design of a microsphere formulation for the intracranial delivery of BMP7 with suitable encapsulation and controlled release properties [11]. We argued that the delivery characteristics of these microspheres resulted from the encapsulation of BMP7 integrated in nanocomplexes with heparin and a polyethylenoxyde derivative. Growth factor release as a complex with heparin help to stabilize these proteins for prolonged action [26], while polyoxyethylene derivatives can protect proteins during encapsulation in hydrophobic matrices [27]. With this technology, the interaction between an anionic heparin/BMP7 complex and a cationic polyethylenoxyde derivative (Tetronic) is further enforced by ionic bonding.

Microscopy analysis showed that the microspheres prepared by the nanocomplex encapsulation method are spherical, regular and many present a hollow interior. Spectroscopic analysis confirmed interaction between the biodegradable PLGA matrix and Tetronic; this good interpolymer compatibility can be related to the efficient encapsulation of the BMP7/heparin/Tetronic complex in the PLGA matrix. Microsphere matrix degraded *in vitro* at 37°C over a period of > 4 weeks, a process triggering BMP7 release [28]. We have previously showed how BMP7 released from the microspheres can stop cell growth in spheres from the U87MG glioma cell line [11], an interesting cell model for screening purposes, but limited for predicting the system's therapeutic potential, and for analyzing the cellular events behind the observed tumor suppressive effects [29]. We addressed these questions here by interrogating a more advance tumor model of primary GBM-TICs cell lines established in our laboratory from surgically resected human specimens of adult GBM patients. The effect of BMP7 was tested not only *in vitro* but also *in vivo*.

We tested the effect of BMP7 *in vivo* by concomitant subcutaneous transplantation of GBM-TICs and BMP7-loaded microspheres into nude mice. This setup is a clinically relevant model for addressing the therapeutic potential of BMP7 since GBM-TIC primary cell lines are able to generate tumors that very closely resemble the original patient's GBM except for the invasive properties (Zahonero and Sánchez, unpublished). We found a net reduction in tumor growth *in vivo* when cells were exposed to the microspheres releasing BMP7. Only microspheres with 0.05% w/w of BMP7 presented a statistically significant reduced volume during the whole experiment and at the endpoint, indicating that low BMP7 doses are not sufficient to counteract tumor growth. Although we began to measure the tumor sizes when the tumors from all the conditions were detectable, we have to point out that 0.05% BMP7 tumors arose later in time than 0.01% BMP7 and control tumors. The lag time between the subcutaneous transplantation and the appearance of a detectable tumor mass was 40 days for control and 0.01% BMP7 tumors, while 0.05% BMP7 tumors were visible approximately 10 days later. Animals were sacrificed at day 63 after tumor injection. Taking into account that the microsphere preparation efficiently releases BMP7 *in vitro* for 40–60 days (Figure 4), it is possible that during the last part of the experiment the tumors were growing in the presence of low amounts of BMP7. We anticipate that more sustained release profiles and larger BMP7 doses may further prevent tumor growth. Microspheres with more prolonged release could be engineered from a similar technological platform by using polymers with longer degradation half-lifes such as poly(ε-caprolactone) [30].

The tumors exposed to 0.05% BMP7 microspheres showed an activation of the canonical BMP signaling pathway. They also presented a downregulation of the proliferation marker *PCNA* and upregulation of the negative cell cycle regulators *CDKN1A* and *CDKN2A*, encoding the cell cycle inhibitory proteins p21 and p16. This would be in line with a previous report showing that BMP7 decreases proliferation of a glioma cell line through cell cycle arrest in the G1 phase, through the modulation of *CDK2, CDKN1A* and p*RB* expression [21]. We also observed a reduction in the expression of stem cell markers, including CD133, SOX2, OLIG2 and NESTIN, in agreement with other groups [6, 7]. Furthermore, we observed a downregulation of *GFAPδ*, a product of alternative splicing variants of *GFAP-alpha,* whose upregulation is directly related to high grade gliomas [31, 32], and which is also a marker of neural stem cells in human [33]. Contrary to previous *in vitro* studies [6, 7], however, we did not find an upregulation of the differentiation markers GFAPα or TUBB3 in the tumors that were exposed to 0.05% BMP7 microspheres. Accordingly, the BMP7-treated tumors were histologically indistinguishable from control tumors, except for their size. Given that the expression of the stem cell markers was reduced, it is possible that BMP7 only triggered an incipient differentiation state of the cells. Another plausible explanation is that BMP7 induced differentiation at early time points, while the BMP7 dose released from the microspheres was high, but that once the BMP7 concentration decreased below a certain threshold, the remaining GBM-TICs, or dedifferentiated cells, repopulated the tumor. This could be in agreement with the absence of differences in the mitotic and necrotic index. This lack of general cytotoxicity further indicates that BMP7 therapy does not affect the bulk of the tumor population and thus validates the selectivity of our strategy towards the GBM-TIC subpopulation.

## MATERIALS AND METHODS

### Materials

Poly(D, L-lactide-co-glycolide) 50:50 Resomer^®^ RG 503 (PLGA) (MW - 35 kDa) was purchased from Boehringer Ingelheim (Germany). Tetronic^®^ 1107 (T1107, HBL - 24 MW - 15 kDa), poly(vinyl alcohol) (PVA) (MW 31–50 kDa) heparin sodium salt grade IA from porcine intestinal mucosa, soybean lecithin, and cotton seed oil were obtained from Sigma Aldrich (Spain). Recombinant human bone morphogenetic protein 7 (BMP7) (pI 8.1 MW - 28.8 kDa), and the kit for its determination were purchased from PeproTech (UK) and the Abnova corporation (Taiwan), respectively. All other solvents and chemicals used were of high grade purity.

### Microsphere preparation

Microspheres were prepared using a nanocomplex encapsulation method previously developed in our laboratory for the efficient entrapment of growth factors [11]. Two different loadings of BMP7 in the microspheres were tested: 0.01% and 0.05% (w/w). Microsphere preparation proceeded as follows: the required amount of BMP7 (2 μg for 0.01% loading or 10 μg for 0.05%) was mixed with the same amount of heparin, both dissolved in a total of 250 μL distilled water. The components in the solution were allowed to interact for 30 min at room temperature with mild stirring. After that time, 50 μL of a solution of Tetronic 1107 in water (50 mg/mL) was added to the mixture, letting it interact with BMP7/heparin complexes for other 30 min. In that way, a nanocomplex of BMP7/heparin/Tetronic 1107 was formed and, without the addition of cryoprotectants, it could be freeze-dried (Virtis Genesis, SP Scientific).

The freeze-drying program was the following: −30°C (1 h) ramped to −40°C (1 h) at 400 mTorr, freeze at −40°C (2 h) at 200 mTorr. Primary drying at −40°C to +20°C at a pressure of 20 mTorr according to the following ramp profile: −40°C (0 – 1080 min), −30°C (1800 min), −20°C (2100 min), −10°C (2220 min), 0°C (2340 min), 10°C (2460 min), and 20°C (2580 min). This was followed by a post-drying phase of 3 h at +20°C.

The resulting freeze-dried cake was resuspended in a solution of 20 mg of poly lactic-co-glycolic (PLGA) in 400 μL of acetonitrile (for a 8:1 PLGA: Tetronic ratio). The organic phase containing the PLGA and the nanocomplexes of BMP7/heparin/Tetronic 1107 was added dropwise to 4 mL of cottonseed oil with 0.5% (w/v) of soybean lecithin under magnetic stirring. To achieve the desired particle size the O/O emulsion droplets were sonicated for 20 s, prior to 10 min of gentle stirring in an extraction hood. Microspheres were hardened by the addition of 2 ml of petroleum ether and this mixture was stirred for 10 more minutes. Finally, microspheres were collected by filtration under vacuum using a nitrocellulose membrane (25 mm, 0.22 μm). The particles were washed with 5 mL of petroleum ether to avoid aggregation. The resulting microspheres were put in 500 μL of PVA 0.5% in water, freeze-dried (same procedure as above) and stored in a dry place under vacuum at room temperature until use.

### Physicochemical characterization of the microspheres

Particle size, morphology and the structure of the microspheres were analyzed using Scanning Electron Microscopy (SEM) (EVO LS15, Zeiss Iberia, Spain). Samples were coated with gold-palladium under vacuum for proper observation. The IR spectra were made in a KBr disk with BRUKER IFS 66v equipment (Bruker Corp.). The perimeter of 150 particles were calculated using the Image J software, available online from the National Institute of Health (USA). The average particle size was expressed as volume mean diameter.

### *In vitro* release studies

Samples comprising 1 mg of microspheres loaded with 0.05% of BMP7 (and controls without BMP7) were incubated with 500 μL of PBS (pH 7.4) containing 1% (w/v) BSA at 37°C and under mild stirring. At several scheduled time points, from 24 h to 4 weeks, microspheres were centrifuged at 7000 g for 10 min at 4°C, and supernatants collected. Antigenically active BMP7, released from the microspheres to the supernatants, was quantified by ELISA according to the manufacturer's instructions.

### Primary cell lines and culture conditions

GBM-TIC cultures were obtained via cell dissociation of human GBM surgical specimens from patients from "Hospital 12 de Octubre" (Madrid, Spain). The tissues were procured after obtaining the patients´ written consent and with the approval of the ethics committee of the hospital. All procedures were performed according to the Spanish national law of biomedical investigation – Law 14/2007, of 3 July (LIB).

Fresh tissue samples were digested mechanically first and enzymatically thereafter, using Accumax (Millipore). Isolated cells were purified using a Ficoll gradient (GE Healthcare) and plated at a density of 50,000 cells per milliliter in a culture medium consisting of Neurobasal (Invitrogen) supplemented with B27 (1:50) (Invitrogen); GlutaMAX (1:100) (Invitrogen); penicillin-streptomycin (1:100, Lonza); 0.4% heparin (Sigma-Aldrich); and 40 ng/mL Epidermal Growth Factor (EGF) and 20 ng/mL of basic Fibroblast Growth Factor (bFGF) (Peprotech). All the primary GBM-TICs cultures used in this study had been proved to form tumors in immunodeficient mice (orthotopic and heterotopic transplants), which resemble the original tumors from the patients ([12] and Zahonero and Sanchez, manuscript in preparation).

### Sphere formation and self-renewal assays

Sphere formation assays were carried out, plating 2.5 cells/μL in 96-well plates (surface area, 0.3 cm^2^) in the presence or absence of BMP7 at 50 ng/mL. Results were analyzed after 7 days by counting the number of spheres (under a phase contrast microscope Zeiss Axiovert 40 CFL) and by measuring their diameters from pictures of random fields captured using a ×10 objective (Image J software).

For the self-renewal assay, spheres from the sphere formation assay (that were grown in the presence or in the absence of BMP7) were dissociated and seeded again at same density (2.5 cells/μL in 96-well plates), in the absence of BMP7. After 7 days, the number of spheres was counted.

### Cell growth

A fraction of the culture at any given passage point, consisting of 250,000 viable cells, was plated and the number of cells generated was quantified at the time of the next passage. To generate the accumulated cell growth curves, the ratio of cell production at each subculturing step was multiplied by the number of cells at the previous point of the curve. This procedure was repeated for each passage.

### Quantitative real time PCR

Total RNA was extracted using a Qiagen miRNeasy kit. RNA samples were reverse transcribed using the PrimeScript^®^ RT-PCR Kit (Takara Bio Inc., Otsu, Japan), and real-time qPCR was carried out using SyBR Premix Ex Taq 2X (Takara Bio Inc., Otsu, Japan). PCR was carried out using the 480 Lightcycler PCR system from Roche and analyzed using the 2 − DDCT method [13]. *GAPDH* served as the house-keeping gene to normalize expression for the genes of interest. Primer sequences for the genes are shown in Table 1 (Supplementary information).

**Table 1:** Primer sequences were designed using Primer Express 3.0 software to obtain similar melting temperatures (~58°C) for both oligonucleotides All quantitative PCR primers were designed between exons with a resultant amplicon of less than 150 bp in length. Primers contained 60% to 80% G/C and were no longer than 26 bp. Lyophilized primers (Sigma-Aldrich) were resuspended in ddH2O to a stock solution of 100 mmol/L. The thermal cycler conditions were as follows: 95°C for 10 minutes, followed by 45 cycles of 95°C for 10 seconds, 60°C for 10 seconds, and 72°C for 10 seconds.

Gene	Forward primer	Reverse primer
*GAPDH*	5′-GCCAAGGTCATCCATGACAACT-3′	5′-AGGGCCATCCACAGTCTTCTG-3′
*BMPR1A*	5′-GAACAGGATGAAGCATTTATTCCAGT-3′	5′-CATCTGAATCTGTTTGGCAATAGTTC-3′
*BMPR1B*	5′-CATGCTTTTGCGAAGTGCAG-3′	5′-CAGGCAACCCAGAGTCATCC-3′
*BMPR2*	5′-GAGTGCCTTTGATGGAACATGAC-3′	5′-ATAATCCGGGTGCTCCTTCA-3′
*BMP7*	5′-CACAACCTGGGCTTACAGCTCT-3′	5′-CTTGAAGAAGGCCACCATGAAG-3′
*SOX2*	5′-ACACCAATCCCATCCACACT-3′	5′-GCAAACTTCCTGCAAAGCTC-3′
*CD133*	5′-TGGATGCAGAACTTGACAACGT-3′	5′-ATACCTGCTACGACAGTCGGTGG-3′
*NESTIN*	5′-CCTCCTGGAGGCTGAGAACTC-3′	5′-AAGGCTGGCACAGGTGTCTC-3′
*OLIG2*	5′-CAGAAGCGCTGATGGTCATA-3′	5′-TCGGCAGTTTTGGGTTATTC-3′
*TUBB3*	5′-TAGTGGAGAACACAGACGAGA-3′	5′-CTGCTGTTCTTACTCTGGATG-3′
*GFAPα*	5′-ACATCGAGATCGCCACCTAC-3′	5′-ATCTCCACGGTCTTCACCAC-3′
*GFAPδ*	5′-ACATCGAGATCGCCACCTAC-3′	5′-CGGCGTTCCATTTACAATCT-3′
*PCNA*	5′-GGAGGCTCTAGCCTGACAAA-3′	5′-CTGAGACTTGCGTAAGGGAA-3′
*CDKN1A*	5′-GTGGCCTTGTCGCTGTCTTG-3′	5′-TCGGCGCTTGGAGTGATAGAA-3′
*CDKN2A*	5′-ACCGGAGGAAGAAAGAGGAG-3′	5′-GCGCTACCTGATTCCAATTC-3′

### Immunoblot analysis

Thirty micrograms of protein per lane were loaded on 12% SDS/PAGE gels and blotted onto PVDF membranes. Membranes were incubated with rabbit polyclonal anti–p-Smad1, 5, 8 (1:1000, Cell Signalling), rabbit polyclonal anti-Smad1, 5, 8 (1:500, Santa Cruz) or mouse monoclonal anti-β-actin (1:5000, Sigma) antibodies, followed by secondary antibodies tagged with HRP (1:50000; GE Healthcare), and bands were visualized using enhanced chemiluminescence and analyzed using ImageJ software. β-actin was used as a loading control.

### Immunocytochemistry

Cell spheres were fixed with 4% paraformaldehide (PFA), washed three times with 0.1% phosphate buffer (PB) and incubated in 0.1% PB containing 10% fetal bovine serum and 0.2% Triton X-100 (blocking buffer) for 1 h. Coverslips were incubated with rabbit polyclonal anti– p-Smad1, 5, 8 primary antibody overnight at 4°C. After three washes in 0.1% PB, immunoreactivity was detected with Cy3-conjugated donkey-anti-rabbit (1:1500; Jackson). Cells were counterstained with 4–6-diamidino-2-phenylindole (DAPI), washed in PB, and mounted. Immunofluorescent samples were analyzed and photographed in a Leica Spectral SP5 confocal microscope.

### Mouse xenografts

Housing and animal experiments were performed according to European Union 86/609/EEC guidelines. Protocols were also approved by the CEyBA animal care and use committee, from Instituto de Salud Carlos III. All the surgical procedures were carried out under general anesthesia with 8 μL/g IP anesthetic solution composed of 10% ketamine (Imalgene) and 9.3% xylazine (Xilagesic) in PBS. Animals were also subcutaneously administered 4 μL/g of a 10% analgesic solution (Metacam) diluted in PBS. After the surgery, mice were woken from anesthesia with 4 μL/g of a 7.7% reverting solution (Antisedan) prepared in PBS.

Heterotopic xenografts were performed in athymic nude *Foxn1nu* mice (Harlan). For control groups, cells (1.5 × 10^6^) were resuspended 1:1 in culture media, mixed with 20 mg of blank microspheres, and subsequently mixed with Matrigel (BD). This cell/microsphere mixture was injected subcutaneously into athymic nude mice. In the case of BMP7 treated tumors, microspheres with a 0.01% and 0.05% (w/w) BMP7 loading were used, for a theoretical dose of 2 and 10 μg. The tumor volume was measured with a caliper when it reached a visible size until the animals were sacrificed.

### Statistics

Data is always presented as mean ± SEM. The number of experiments performed in independent cultures/animals (n) is indicated. A 2-tailed Student's *t* test was carried out for statistical analysis of the *in vitro* studies to compare group means. For comparisons between relative values, these were first normalized by using an arcsin transformation. *p < 0.05, **p < 0.01, ***p < 0.001. In all analyses, the null hypothesis was rejected at *p* > 0.05.

## CONCLUSIONS

In this work we have validated the therapeutic potential of BMP7-loaded controlled release microspheres as a specific treatment against GBM-TICs. Primary tumors treated with BMP7-loaded microspheres showed delayed tumor growth and lower tumor final volume, which could be correlated with the activation of the BMP canonical pathway. More importantly, these tumors showed a modified gene expression signature with loss of stem cell traits, typical of tumors with poor prognosis, and overexpression of cell cycle regulators. We propose that the combination of these GBM-TIC specific treatments with conventional cytoreductive chemo- and radiotherapies might result in improved survival.
